# Supplementary material for: One-pot biocatalytic route from cycloalkanes to α,ω‐dicarboxylic acids by designed Escherichia coli consortia
Source: Nat Commun. 2020 Oct 7;11:5035. doi: 10.1038/s41467-020-18833-7 (PMC7542165; doi:10.1038/s41467-020-18833-7)
Supplement: Supplementary file 1 — Supplementary Information [file 41467_2020_18833_MOESM1_ESM.pdf]

Supplementary Information

One-pot biocatalytic route from cycloalkanes to α, ω - dicarboxylic acids by designed *Escherichia coli* consortia

Wang, Zhao, Li et al.

Supplementary Methods

Stability test of E. coli cell modules S4

Bacterial viability assay S4

Western blot analysis S4

*Scale-up in bioreactor for converting CH **1b** or CHOL **2b** to AA **7b**.* S5

Supplementary Tables

***Supplementary Table 1**. The information of recombinant enzymes for western blot analysis* S6

***Supplementary Table 2.** Concentration of P450$_{BM3}$.* S6

***Supplementary Table 3**. Comparison of current method with other reported bio-based routes using E. coli as catalyst.* S7

***Supplementary Table 4.** Strains and plasmids* S8

***Supplementary Table 5.** Oligonucleotide sequences* S9

***Supplementary Table 6.** Synthetic gene sequences* S11

Supplementary Figures

***Supplementary Figure 1.** AA production using E. coli (M2E_M3J) or a mixture of E. coli (M2E) and E. coli (M3J).* S15

***Supplementary Figure 2.** AA production using E. coli (M12A_M3J) or a mixture of E. coli (M1D), E. coli (M2E) and E. coli (M3J).* S15

***Supplementary Figure 3.** SDS-PAGE analysis of whole-cell proteins of Module 3 expressed in E. coli.* S16

***Supplementary Figure 4.** Overrall concept for producing adipic acid based on biorenewable feedstock.* S16

***Supplementary Figure 5.** SDS-PAGE analysis of whole-cell proteins of Module 2 expressed in E. coli.* S17

***Supplementary Figure 6.** SDS-PAGE analysis of whole-cell proteins of Module 1 expressed in E. coli.* S17

***Supplementary Figure 7.** Western blot analysis of recombinant enzyme expressions.* S18

***Supplementary Figure 8.** Scale-up in bioreactor for converting CHOL **2b** to AA **7b**.* S19

Supplementary Figure 9.** Images of the 1-L fermenter and product AA **7b S19

***Supplementary Figure 10.** Reaction time course with module 1 and EC1_2_3 as catalysts.* S20

***Supplementary Figure 11.** Stability assay of each E. coli cell module.* S20

***Supplementary Figure 12.** Bacterial viability assay during the biocatalytic reaction with EC1_2_3.* S21

***Supplementary Figure 13.** Scale-up in bioreactor for converting CH **1b** to AA **7b**.* S21

***Supplementary Figure 14.** Performance of each cell module at varying substrate concentrations.* S22

***Supplementary Figure 15.** GC analysis of reaction mixture from E. coli consortiums catalyzed conversion of either **2b** or **1b** to **7b**.* S23

***Supplementary Figure 16.** GC analysis of reaction mixtures from E. coli consortium 2_3 catalyzed conversion of **2a**, **2c**, **2d** to **7a**, **7c**, **7d**.* S24

***Supplementary Figure 17.** GC analysis of reaction mixtures from E. coli consortium* S25

*1_2_3 catalyzed converison of **1a**, **1c**, **1d** to 7**a**, 7**c**, 7**d**.*
Supplementary Figure 18. *GC-MS analysis of α, ω-dicarboxylic acids 7**a**-**b**.* S26
Supplementary Figure 19. *GC-MS analysis of α, ω-dicarboxylic acids 7**c**-**d**.* S27
Supplementary Figure 20. *NMR spectra of 7**a**-**b**.* S28
Supplementary Figure 21. *NMR spectra of 7**c**-**d**.* S29
Supplementary References S30

Supplementary Methods

Stability test of *E. coli* cell modules. The stability of each cell module was evaluated by determining their catalytic performance after pretreatment for different periods of time (0-24 h). 4 mL each cell module with 8 g CDW L^{-1} was incubated at 25°C, 200 r.p.m, and after a certain time (0, 3, 6, 12 and 24 h), the substrate CH **1b**, CHOL **2b**, or CL **3b** (final concentration: 100 mM) was added to start the reaction for cell module 1, module 2 and module 3, respectively. At 10 h of reaction, the corresponding products were determined by GC analysis.

Bacterial viability assay. A LIVE/DEAD® BacLight™ Bacterial Viability kit was utilized for assessing bacterial membrane integrity during the reaction. The bacteria with intact cell membranes were labeled green-fluorescence *via* nucleic acid stain SYTO 9, whereas bacteria with damaged membrane were dyed fluorescent red with propidium iodide. The excitation/emission maxima for these stains are about 480/500 nm for SYTO 9 and 490/635 nm for propidium iodide.

Bacterial viability tests were performed in triplicate as described by the manufacturer. Briefly, overnight bacterial culture was centrifuged, resuspended in potassium phosphate buffer (0.2 M, pH 8.0). The suspension was then treated with 0.85% NaCl or 70% isopropyl alcohol for 1 h to obtain viable or non-viable bacteria, respectively. The live or dead cells were adjusted to 2×10^8 bacteria per mL. Different portions of live- and dead-cell suspension were mixed and both dyes were added in a ratio of 3‰. Similarly, samples (with or without CH **1b**) at different time point (0, 3, 6, 12, 24 and 30 h) were adjusted to 2×10^8 bacteria per mL and mixed with both dyes with the same ratio. The mixture was incubated at room temperature in the dark for 15 min and then determined with fluorescence microplate reader SpectraMax. The percentage of live bacteria was proportional to the ratio of green to red fluorescence emission.

Western blot analysis. Five recombinant enzymes (P450, ADH1, ADH2, ALDH and lactonase) were expressed with His-tag, and other three enzymes (GDH, BVMO and NOX) were expressed with Flag-tag (more details in Supplementary Table 1). The recombinant *E. coli* cells were collected, washed twice and resuspended in potassium phosphate buffer (0.2 M, pH 8.0) to an optical density (600 nm) of 20. After sonication and centrifugation (13, 000 × *g*, 4°C for 15 min), the supernatant fraction was collected and mixed with 5 × loading buffer (250 mM Tris-HCl with pH 6.8, 10% SDS, 50% glycerol, 0.5% bromphenol blue and 5% β-mercaptoethanol), then boiled for 10 min.

Proteins samples were separated using 10% SDS-PAGE, and then transferred onto PVDF membranes. Afterwards, the membranes were incubated with 5% non-fat milk in PBST (PBS with 0.05% tween) overnight for blocking. The anti-His mouse monoclonal antibody or anti-Flag mouse monoclonal antibody was diluted by a factor of 3000 in PBST buffer containing 5% non-fat milk. The membranes were incubated in this solution at 37℃ for 1 h, followed by washing the membranes 5 times with PBST. Then, the membranes were incubated with goat anti mouse monoclonal secondary antibody (diluted at 1:5000) at room temperature for 1 h. Again, the membranes were washed 5 times with PBST, and chemiluminescence reagent was used to develop the bands, which were visualized by Amersham Imager 600 (GE Healthcare Life Sciences). Meanwhile, we detected the concentration of P450 with the method of CO-binding difference spectra as previously described by Omura T and Sato R[1].

Scale-up in bioreactor for converting CH 1b or CHOL 2b to AA 7b. The biotransformation of CH **1b** or CHOL **2b** to AA **7b** was scaled up in 1-L fermenters (Baoxing Biotech, Shanghai, China) using *E. coli* consortia. The whole-cell biocatalysts were cultured as described in the main text and suspended in 400 mL potassium phosphate buffer (0.2 M, pH 8.0). For the EC2_3-catalyzed reaction, 50 mM CHOL **2b** was converted with EC2_3 (final CDW was 16 g L^{-1}, module 2: module 3=2:1). In the case of EC1_2_3-catalyzed reactions, two experiments were carried out: (a) EC1_2_3 with ratio of 2:1:2 (module 1: module 2: module 3, final CDW was 12 g L^{-1}, glucose concentration was 0.04 g mL^{-1}) was used to convert 200 mM CH **1b** with 15 g resin; (b) CH **1b** (100 mM) was first catalyzed by module 1 (4 g CDW L^{-1} with 0.04 g mL^{-1} glucose), and then module 2 (8 g CDW L^{-1}) and module 3 (4 g CDW L^{-1}) were added at 22 h of reaction. All reactions were at 25°C, 500 r.p.m in 1-L fermenters, and pH was maintained around 8.0 by adding 10 M NaOH. Samples were taken at appropriate intervals and prepared for GC analysis as described for modular cell 2 converting **2b** to **4b**.

Supplementary Tables

Supplementary Table 1. The information of recombinant enzymes for western blot analysis. The theoretical molecular weights in kDa and locations of His- or Flag-tag are given in the brackets.

Cell module	His-tagged enzymes	Flag-tagged enzymes
Module 1	P450 (119, N-terminal)	GDH (29, N-terminal)
Module 2	ADH1 (28, N-terminal)	BVMO (62, N-terminal)
Module 3	ADH2 (38, N-terminal), ALDH (53, C-terminal), Lactonase (34, N-terminal)	NOX (50, C-terminal)
EC2_3	ADH1 (28, N-terminal), ADH2 (38, N-terminal), ALDH (53, C-terminal), Lactonase (34, N-terminal)	BVMO (62, N-terminal), NOX (50, C-terminal)
EC1_2_3	P450 (119, N-terminal), ADH1 (28, N-terminal), ADH2 (38, N-terminal), ALDH (53, C-terminal), Lactonase (34, N-terminal)	GDH (29, N-terminal), BVMO (62, N-terminal), NOX (50, C-terminal)

Supplementary Table 2. Concentration of $P450_{BM3}$.

Cell module	$P450_{BM3}$ concentration (μM)
Module 1	2.20 ± 0.06
EC1_2_3	ND[a]

[a] Not detectable.

Source data are provided as a Source Data file.

Supplementary Table 3. Comparison of current method with other reported bio-based routes using *E. coli* as catalyst.

Products	Substrates	Methods	Purification	Titer ($g\ L^{-1}$)	References
**Glutaric acid**	Glucose	Fermentation (Growing cell)	Extraction, ion exchange, recrystallization	0.82	[2]
	Cyclopentane/cyclopentanol	Biocatalysis (Resting cell)	Extraction	1.6/6.3	This study
**Adipic acid**	Glucose	Fermentation (Growing cell)	Extraction, ion exchange, recrystallization	68.0	[3]
	Cyclohexane/cyclohexanol/ε-caprolactone	Biocatalysis (Resting cell)	Extraction recrystallization （optional）	4.5/6.7/ 66.0	This study
**Pimelic acid**	-	-	-	-	N.A.
	Cycloheptane/cycloheptanol	Biocatalysis (Resting cell)	Extraction	3.2/7.7	This study
**Suberic acid**	Glycerol	Fermentation (Growing cell)	Extraction, ion exchange, recrystallization	0.254	[4]
	Cyclooctane/cyclooctanol	Biocatalysis (Resting cell)	Extraction	1.1/7.3	This study

N.A.: Not available

Supplementary Table 4. Strains and plasmids

Strain	Description	Source
E. coli BL21 (DE3)	*F^-ompT gal dcm lon hsdS$_B$ (r_B^- m_B^-) λ(DE3 [lacI lacUV5-T7 gene 1 ind1 sam7 nin5])*	Invitrogen

Plasmid	Description	Source
M3A	pRSFDuet-1 carrying ADH2 and ALDH	This study
M3B	pETDuet-1 carrying ADH2 and ALDH	This study
M3C	pRSFDuet-1 carrying Lactonase	This study
M3D	pETDuet-1 carrying Lactonase	This study
M3E	pRSFDuet-1 carrying Lactonase and NOX	This study
M3F	pETDuet-1 carrying Lactonase and NOX	This study
M3G	pRSFDuet-1 carrying ADH2, ALDH and Lactonase	This study
M3H	pETDuet-1 carrying ADH2, ALDH and Lactonase	This study
M3I	pRSFDuet-1 carrying ADH2, ALDH, Lactonase and NOX	This study
M3J	pETDuet-1 carrying ADH2, ALDH, Lactonase and NOX	This study
M2A	pRSFDuet-1 carrying ADH1	This study
M2B	pETDuet-1 carrying ADH1	This study
M2C	pRSFDuet-1 carrying BVMO	This study
M2D	pETDuet-1 carrying BVMO	This study
M2E	pRSFDuet-1 carrying ADH1 and BVMO	This study
M2F	pETDuet-1 carrying ADH1 and BVMO	This study
M2G	pRSFDuet-1 carrying BVMO and ADH1	This study
M2H	pETDuet-1 carrying BVMO and ADH1	This study
M1A	pRSFDuet-1 carrying $P450_{BM3}$A82F	This study
M1B	pRSFDuet-1 carrying $P450_{BM3}$A82F/A328F	This study
M1C	pRSFDuet-1 carrying $P450_{BM}$19A12	This study
M1D	pRSFDuet-1 carrying $P450_{BM}$19A12 and GDH	This study
M12A	pRSFDuet-1 carrying $P450_{BM}$19A12, GDH, ADH1 and BVMO	This study
pETDuet-1	Double T7 promoters, pBR322 ori, Amp^R	Novagen
pRSFDuet-1	Double T7 promoters, RSF ori, Kn^R	Novagen

Supplementary Table 5. Oligonucleotide sequences

Name	Sequence (5' →3')
ADH2_homologous seq-Fwd	GCCATCACCATCATCACCACCATTGTTATTGCGTTACCCATCATGG
ADH2_RBS-Rev	GATATATCTCCTTAGGTACCTTAGTTCTCGTGCATCAGAACGATACG
Lactonase_homologous seq-Fwd	GCCATCACCATCATCACCACACCAATATTAGCGAAACCCTGAGCAC
Lactonase_homologous seq-Rev	GCTCGAATTCGGATCCTGGCTTATTCCAGGGCTTTCTGATACCATGCTG
Lactonase _RBS-Rev	GATATATCTCCTTAGGTACCTTATTCCAGGGCTTTCTGATACCATGCTG
RBS_ Lactonase-Fwd	GGTACCTAAGGAGATATATCATGACCAATATTAGCGAAACCCTGAGC
ALDH_RBS-Rev	GATATATCTCCTTAGGTACCTTAGTTCAGCTGGGTGATAAATTTGGTG
RBS_ ALDH-Fwd	GGTACCTAAGGAGATATATCATGAACTATCCGAATATTCCGCTGTATATTAACG
ALDH_ homologous seq-Rev	GCTCGAATTCGGATCCTGGCTTAGTTCAGCTGGGTGATAAATTTGGTG
NOX_ homologous seq-Rev	GCTCGAATTCGGATCCTGGCTTATTCCGTCACTTTTTCAGCCGCATGAG
RBS_NOX-Fwd	GGTACCTAAGGAGATATATCATGAAAGTTATCGTAATTGGTTGTACTCATGCCG
BVMO_homologous seq-Fwd	CACCATCATCACCACATGTCACAAAAAATGGATTTTGATGCTATCGTGATTG
BVMO_homologous seq-Rev	GAATTCGGATCCTGGCTTAGGCATTGGCAGGTTGCTTGATATC
BVMO_RBS-Rev	GATATATCTCCTTAGGTACCTTAGGCATTGGCAGGTTGCTTGATATCTG
RBS_BVMO-Fwd	GGTACCTAAGGAGATATATCATGTCACAAAAAATGGATTTTGATGCTATCGTG
ADH1_homologous seq-Fwd	CACCATCATCACCACATGAGCAATCGTCTGGATGGTAAAGTTG
ADH1_homologous seq-Rev	AATTCGGATCCTGGCTTACTGTGCGGTATAACCACCATCCAC
ADH1_RBS-Rev	GATATATCTCCTTAGGTACCTTACTGTGCGGTATAACCACCATCCAC
RBS_ADH1-Fwd	GGTACCTAAGGAGATATATCATGAGCAATCGTCTGGATGGTAAAGTTG
19A12_homologous seq-Fwd	CACCATCATCACCACGCAATTAAAGAAATGCCTCAGCCAAAAACG
19A12_homologous seq-Rev	AATTCGGATCCTGGCTTACCCAGCCCACACGTCTTTTGC
19A12_RBS-Rev	GATATATCTCCTTAGGTACCTTACCCAGCCCACACGTCTTTTGC
RBS_GDH-Fwd	GGTACCTAAGGAGATATATCATGTATACAGATTTAAAAGATAAAGTAGTAGTAAT TACAGGTGGATC
GDH_RBS-Rev	GATATATCTCCTTAGGTACCTTATCCGCGTCCTGCTTGGAATG
GDH_ homologous seq-Rev	GCTCGAATTCGGATCCTGGCTTATCCGCGTCCTGCTTGGAATG
pRSFDuet-1_homologous seq-Fwd	GCCAGGATCCGAATTCGAGCTC
pRSFDuet-1_homologous seq-Rev	GTGGTGATGATGGTGATGGCTGCTG
pETDuet-1_homologous seq-Fwd	GCCAGGATCCGAATTCGAGCTC
pETDuet-1_homologous seq-Rev	GTGGTGATGATGGTGATGGCTGCTG
Flag-GDH-Fwd	GATTATAAAGATGATGATGATAAATATACAGATTTAAAAGATAAAGTAGTAG
Flag-RBS-P450- Rev	TTTATCATCATCATCTTTATAATCCATGATATATCTCCTTAGGTACCTTACC
Flag-BVMO- Fwd	GATTATAAAGATGATGATGATAAATCACAAAAAATGGATTTTGATGCTATCG
Flag-RBS-LBADH- Rev	TTTATCATCATCATCTTTATAATCCATGATATATCTCCTTAGGTACCTTACTGTG
ChnE-C6His- Fwd	CATCACCATCATCACCACTAAGCCAGGATCCGAATTCGAGCTC
C6His-ChnE- Rev	GTGGTGATGATGGTGATGGTTCAGCTGGGTGATAAATTTGGTGCG
CFlag-Z- Fwd	GATTATAAAGATGATGATGATAAATAAGCCAGGATCCGAATTCGAGCTCG
CFlag-NOX- Rev	TTTATCATCATCATCTTTATAATCTTCCGTCACTTTTTCAGCCGCATG
RBS-6His-M- Fwd	GGTACCTAAGGAGATATATCATGGGCAGCAGCCATCACC
CFlag-NOX- Rev	CATGCGGCTGAAAAAGTGACGGAAGATTATAAAGATGATGATGATAAA

NOX-CFlag- Fwd	GATTATAAAGATGATGATGATAAATAAGCCAGGATCCGAATTCGAGCTCG
ChnE-6His-RBS- Rev	GATATATCTCCTTAGGTACCTTAGTGGTGATGATGGTGATGGTTCAG
pET/pRSDuetI - Fwd	GCCAGGATCCGAATTCGAGCTC

Supplementary Table 6. Synthetic gene sequences

Glucose dehydrogenase (GDH) from *Bacillus megaterium*

ATGTATACAGATTTAAAAGATAAAGTAGTAGTAATTACAGGTGGATCAACAGGTTTAG
GACGCGCAATGGCTGTTCGTTTCGGTCAAGAAGAAGCAAAAGTTGTTATTAACTATT
ACAACAATGAAGAAGAAGCTTTAGATGCGAAAAAAGAAGTAGAAGAAGCAGGCGG
ACAAGCAATCATCGTTCAAGGCGACGTAACAAAAGAAGAAGATGTTGTAAACCTTG
TTCAAACAGCTATTAAAGAATTCGGTACATTAGACGTTATGATTAATAACGCTGGTGT
TGAAAACCCAGTTCCTTCTCATGAGTTATCTTTAGACAACTGGAATAAAGTTATTGAT
ACAAACTTAACAGGTGCATTCTTAGGAAGCCGTGAAGCAATCAAATATTTTGTTGAA
AACGACATTAAAGGAAACGTTATTAACATGTCTAGTGTTCATGAAATGATTCCTTGGC
CATTATTTGTTCATTACGCAGCAAGTAAAGGCGGTATGAAACTAATGACGGAAACAT
TGGCTCTTGAATATGCGCCAAAAGGTATCCGCGTAAATAACATTGGACCAGGTGCGA
TGAACACACCAATTAACGCAGAGAAATTTGCAGATCCTGTACAACGTGCAGACGTA
GAAAGCATGATTCCAATGGGTTACATCGGTAAACCAGAAGAAGTAGCAGCAGTTGC
AGCATTCTTAGCATCATCACAAGCAAGCTATGTAACAGGTATTACATTATTTGCTGATG
GTGGTATGACGAAATACCCATCATTCCAAGCAGGACGCGGATAA

Alcohol dehydrogenases (ADH1) from *Acinetobacter* sp. NCIMB9871

ATGAGCAATCGTCTGGATGGTAAAGTTGCAATTATTACCGGTGGCACCTTAGGTATTG
GTCTGGCAATTGCAACCAAATTTGTTGAAGAGGGTGCCAAAGTTATGATTACCGGTC
GTCATAGTGATGTTGGTGAAAAAGCAGCAAAAAGCGTTGGTACACCGGATCAGATT
CAGTTTTTTCAGCATGATAGCAGTGATGAAGATGGTTGGACCAAACTGTTTGATGCA
ACCGAAAAAGCATTTGGTCCGGTTAGCACCCTGGTTAATAATGCAGGTATTGCAGTG
AATAAGAGCGTTGAAGAAACCACCACCGCAGAATGGCGTAAACTGCTGGCAGTTAA
TCTGGATGGCGTTTTTTTGGTACACGTCTGGGTATTCAGCGCATGAAAAACAAAGG
TCTGGGTGCAAGCATTATCAACATGAGCAGCATTGAAGGTTTTGTTGGTGATCCGAG
CCTGGGTGCATATAATGCAAGCAAAGGTGCAGTTCGTATTATGAGCAAAAGCGCAGC
ACTGGATTGTGCACTGAAAGATTATGATGTTCGTGTGAATACCGTTCATCCGGGTTAT
ATCAAAACACCGCTGGTTGATGATCTGCCTGGTGCCGAAGAAGCAATGAGCCAGCG
TACAAAAACCCCGATGGGTCATATTGGTGAACCGAATGATATTGCCTATATCTGTGTT
TATCTGGCCAGCAACGAAAGTAAATTTGCAACCGGTAGCGAATTTGTTGTGGATGGT
GGTTATACCGCACAGTAA

Baeyer-Villiger monooxygenase (BVMO) from *Acinetobacter* sp. NCIMB9871

ATGTCACAAAAAATGGATTTTGATGCTATCGTGATTGGTGGTGGTTTTGGCGGACTTT

ATGCAGTCAAAAAATTAAGAGACGAGCTCGAACTTAAGGTTCAGGCTTTTGATAAA
GCCACGGATGTCGCAGGTACTTGGTACTGGAACCGTTACCCAGGTGCATTGACGGAT
ACAGAAACCCACCTCTACTGCTATTCTTGGGATAAAGAATTACTACAATCGCTAGAA
ATCAAGAAAAAATATGTGCAAGGCCCTGATGTACGCAAGTATTTACAGCAAGTGGCT
GAAAAGCATGATTTAAAGAAGAGCTATCAATTCAATACCGCGGTTCAATCGGCTCAT
TACAACGAAGCAGATGCCTTGTGGGAAGTCACCACTGAATATGGTGATAAGTACACG
GCGCGTTTCCTCATCACTGCTTTAGGCTTATTGTCTGCGCCTAACTTGCCAAACATCA
AAGGCATTAATCAGTTTAAAGGTGAGCTGCATCATACCAGCCGCTGGCCAGATGACG
TAAGTTTTGAAGGTAAACGTGTCGGCGTGATTGGTACGGGTTCCACCGGTGTTCAGG
TTATTACGGCTGTGGCACCTCTGGCTAAACACCTCACTGTCTTCCAGCGTTCTGCAC
AATACAGCGTTCCAATTGGCAATGATCCACTGTCTGAAGAAGATGTTAAAAAGATCA
AAGACAATTATGACAAAATTTGGGATGGTGTATGGAATTCAGCCCTTGCCTTTGGCCT
GAATGAAAGCACAGTGCCAGCAATGAGCGTATCAGCTGAAGAACGCAAGGCAGTTT
TTGAAAAGGCATGGCAAACAGGTGGCGGTTTCCGTTTCATGTTTGAAACTTTCGGTG
ATATTGCCACCAATATGGAAGCCAATATCGAAGCGCAAAATTTCATTAAGGGTAAAAT
TGCTGAAATCGTCAAAGATCCAGCCATTGCACAGAAGCTTATGCCACAGGATTTGTA
TGCAAAACGTCCGTTGTGTGACAGTGGTTACTACAACACCTTTAACCGTGACAATGT
CCGTTTAGAAGATGTGAAAGCCAATCCGATTGTTGAAATTACCGAAAACGGTGTGAA
ACTCGAAAATGGCGATTTCGTTGAATTAGACATGCTGATACTGGCCACAGGTTTTGAT
GCCGTCGATGGCAACTATGTGCGCATGGACATTCAAGGTAAAAACGGCTTGGCCATT
AAAGACTACTGGAAAGAAGGTCCGTCGAGCTATATGGGTGTCACCGTAAATAACTAT
CCAAACATGTTCATGGTGCTTGGACCGAATGGCCCGTTTACCAACCTGCCGCCATCA
ATTGAATCACAGGTGGAATGGATCAGTGATACCATTCAATACACGGTTGAAAACAAT
GTTGAATCCATTGAAGCGACAAAAGAAGCGGAAGAACAATGGACTCAAACTTGCGC
CAATATTGCGGAAATGACCTTATTCCCTAAAGCGCAATCCTGGATTTTTGGTGCGAAT
ATCCCGGGCAAGAAAAACACGGTTTACTTCTATCTCGGTGGTTTAAAAGAATATCGC
AGTGCGCTAGCCAACTGCAAAAACCATGCCTATGAAGGTTTTGATATTCAATTACAA
CGTTCAGATATCAAGCAACCTGCCAATGCCTAA

Alcohol dehydrogenases (ADH2) from *Acinetobacter* sp. NCIMB9871

ATGCATTGTTATTGCGTTACCCATCATGGTCAGCCGCTGGAAGATGTTGAAAAAGAA
ATTCCGCAGCCGAAAGGCACCGAAGTTCTGCTGCATGTTAAAGCAGCAGGTCTGTG
TCATACCGATCTGCATCTGTGGGAAGGTTATTATGATTTAGGTGGTGGTAAACGTCTG
AGCCTGGCAGATCGTGGTCTGAAACCGCCTCTGACACTGAGCCATGAAATTACCGG
TCAGGTTGTTGCAGTTGGTCCGGATGCAGAAAGCGTTAAAGTTGGTATGGTTAGCCT
GGTTCATCCGTGGATTGGTTGTGGTGAATGTAATTATTGTAAACGCGGTGAAGAAAA

CCTGTGTGCAAAACCGCAGCAGCTGGGTATTGCAAAACCTGGTGGTTTTGCAGAAT
ACATTATTGTTCCGCATCCGCGTTATCTGGTTGATATTGCAGGTCTGGATCTGGCCGA
AGCAGCACCGCTGGCATGTGCCGGTGTTACCACCTATAGCGCACTGAAAAAATTCGG
TGATCTGATTCAGAGCGAACCGGTTGTTATTATTGGTGCCGGTGGTCTGGGTCTGATG
GCACTGGAACTGCTGAAAGCAATGCAGGCAAAAGGTGCAATTGTTGTGGATATCGA
TGATAGCAAACTGGAAGCAGCCCGTGCAGCCGGTGCACTGAGCGTGATTAATAGCC
GTAGCGAAGATGCAGCACAGCAGCTGATTCAGGCCACCGATGGTGGTGCACGTCTG
ATTCTGGACCTGGTTGGTAGCAATCCGACACTGAGTCTGGCACTGGCAAGCGCAGC
ACGTGGTGGTCATATTGTTATTTGTGGCCTGATGGGTGGTGAAATCAAACTGAGCATT
CCGGTTATTCCGATGCGTCCGCTGACCATTCAGGGTAGCTATGTTGGCACCGTTGAA
GAACTGCGTGAACTGGTTGAGCTGGTTAAAGAAACCCATATGAGCGCAATTCCGGT
GAAAAAACTGCCGATTAGCCAGATTAATAGTGCCTTTGGCGATCTGAAAGATGGTAA
TGTTATTGGTCGTATCGTTCTGATGCACGAGAACTAA

Aldehyde dehydrogenase (ALDH) from *Acinetobacter* sp. NCIMB9871

ATGAACTATCCGAATATTCCGCTGTATATTAACGGCGAATTTCTGGATCATACCAATCG
TGATGTGAAAGAAGTGTTTAACCCGGTTAACCATGAATGCATTGGTCTGATGGCATG
TGCAAGCCAGGCAGATCTGGATTATGCACTGGAAAGCAGCCAGCAGGCATTTCTGC
GTTGGAAAAAAACCAGTCCGATTACACGTAGCGAAATTCTGCGTACCTTTGCAAAA
CTGGCACGTGAAAAAGCAGCAGAAATTGGTCGCAATATTACCCTGGATCAGGGCAA
ACCGCTGAAAGAAGCAATTGCCGAAGTTACCGTTTGTGCAGAACATGCAGAATGGC
ATGCAGAAGAATGTCGTCGTATTTATGGTCGTGTTATTCCGCCTCGTAATCCGAATGT
TCAGCAGCTGGTTGTTCGTGAACCGCTGGGTGTTTGTCTGGCATTTAGCCCGTGGAA
TTTTCCGTTTAATCAGGCCATTCGTAAAATCAGCGCAGCAATTGCAGCAGGTTGTAC
CATTATTGTTAAAGGTAGCGGTGATACCCCGAGCGCAGTTTATGCAATTGCCCAGCTG
TTTCATGAAGCAGGTCTGCCGAATGGTGTTCTGAATGTTATTTGGGGTGATAGCAACT
TCATCAGCGACTATATGATTAAAAGCCCGATCATCCAGAAAATCAGCTTTACCGGTAG
CACACCGGTTGGTAAAAAACTGGCCAGCCAGGCAAGCCTGTATATGAAACCGTGTA
CCATGGAATTAGGTGGTCATGCACCGGTTATTGTTTGTGATGATGCAGATATTGATGC
AGCCGTTGAACATCTGGTTGGTTACAAATTTCGTAATGCAGGTCAGGTTTGTGTTAG
CCCGACACGTTTTTATGTTCAAGAGGGCATCTATAAAGAGTTTAGCGAAAAAGTTGT
TCTGCGTGCCAAGCAGATTAAAGTTGGTTGTGGTCTGGATGCAAGCAGCGATATGGG
TCCGCTGGCACAGGCACGTCGTATGCATGCAATGCAGCAGATCGTTGAAGATGCAGT
TCATAAAGGTAGTAAACTGCTGTTAGGTGGCAACAAGATTAGCGATAAAGGCAACTT
TTTTGAACCGACCGTTCTGGGTGATCTGTGTAATGATACCCAGTTTATGAACGATGAA
CCGTTTGGTCCGATTATCGGTCTGATTCCGTTTGATACCATTGATCATGTTCTGGAAG

AAGCAAATCGTCTGCCGTTTGGCCTGGCAAGCTATGCATTTACCACCAGTAGCAAAA<br>ATGCACACCAGATTAGCTATGGTCTGGAAGCAGGTATGGTTAGCATTAACCATATGGG<br>TTTAGCACTGGCAGAAACCCCGTTTGGTGGTATTAAAGATAGTGGTTTTGGTAGCGA<br>AGGTGGCATTGAAACCTTTGATGGTTATCTGCGCACCAAATTTATCACCCAGCTGAA<br>CTAA
**Lactonase from *Rhodococcus* sp. HI-31**
ATGACCAATATTAGCGAAACCCTGAGCACCGCACCTGGTGGTGCAGCAGGTCCGGA<br>TGTTCTGCGTGATCTGTATGCAGATTGGAGCGAAATTATGGCAGCAACACCGGATCT<br>GACCATTCGTCTGCTGCGTAGCCTGTTTGATGAATGGCATCAGCCGACCGTTGAACC<br>GGAAGGTGTTACCTATCGTGAAGAAACCGTTGGTGGTGTTCCTGGTATTTGGTGTCT<br>GCCGCAGGGTGCAGATGGTAGCAAAGTTCTGCTGTATACCCATGGTGGTGGTTTTGC<br>AGTTGGTAGCGCAGCAAGCCATCGTAAACTGGCAGGTCATGTTGCAAAAGCACTGG<br>GTGCCGTTGGTTTTGTTCTGGATTATCGTCGTGCACCGGAATTTCAGCATCCGGCACA<br>GATTGAAGATGGTGTTGCAGCATTTGATGCACTGGTTGCAAATGGTATTGCACCGCA<br>GGATATTACCACCATTGGTGATAGTGCCGGTGGTAATCTGGCAGTTGCAATTGCCCTG<br>AGCCTGCGTGAACAGGGTAAACAAGGTCCGGGTAGCGTTATTGCATTTAGCCCGTGG<br>CTGGATATGGAAAATAAAGGTGAAACCCTGGCCACCAATAATGATACCGATGCACTG<br>ATTACACCGGAACTGCTGGAAGGCATGATTGCCGGTGTGCTGGGTGATACCATTGAT<br>CCGAAAACACCGCTGGCAAATCCGCTGTATGCCGATTTACCGGTTTTCCGCGTCTG<br>TATATCACCGCAGGTAGCGTTGAAAGCCTGCTGGATAATGCAACCCGTCTGGAAAAA<br>TTAGCAGCATCTGCCGGTGTTGATGTTACCCTGAGTATTGGTGAAGGTCAGCAGCAT<br>GTTTATCCGTTTCTGGCAGGCCGTAGCGCACTGGTGGATGATGAATTTGCAAAGCTG<br>GCAGCATGGTATCAGAAAGCCCTGGAATAA

Supplementary Figures

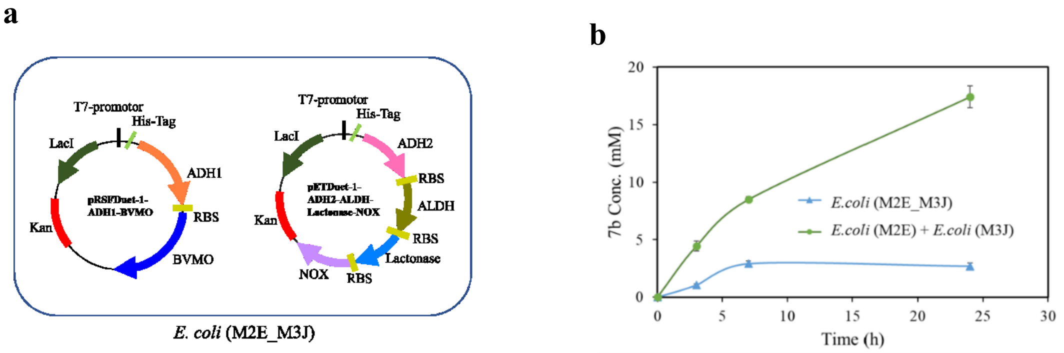

Supplementary Figure 1. AA production using *E. coli* (M2E_M3J) or a mixture of *E. coli* (M2E) and *E. coli* (M3J). a Construction maps of M2E and M3J plasmids *in E. coli* (M2E_M3J) strain. **b** Time course of AA **7b** production from cyclohexanol **2b** with resting cells of *E. coli* (M2E_M3J) or a mixture of *E. coli* (M2E) and *E. coli* (M3J) (ratio was 1:1). The substrate concentration was 50 mM, and final CDW of cells for two reactions was 16 g L^{-1}. Green line: AA concentration using *E. coli* (M2E_M3J) as catalyst; Blue line: AA concentration using the mixture of *E. coli* (M2E) and *E. coli* (M3J) as catalyst. Data are presented as mean value ± SD (standard deviations) of three biological replicates. Source data are provided as a Source Data file.

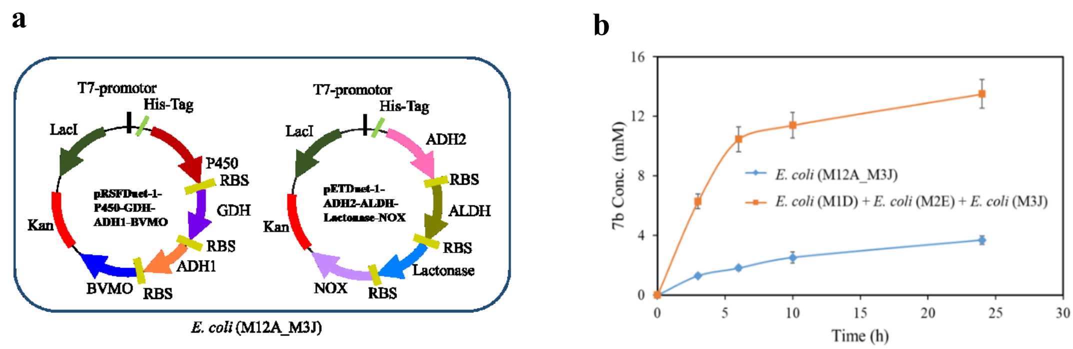

Supplementary Figure 2. AA production using *E. coli* (M12A_M3J) or a mixture of *E. coli* (M1D), *E. coli* (M2E) and *E. coli* (M3J). a Construction maps of M12A and M3J plasmids *in E. coli* (M12A_M3J) strain. **b** Time course of AA **7b** production from cyclohexane **2b** with resting cells of *E. coli* (M12A_M3J) or the mixture of *E. coli* (M1D), *E. coli* (M2E) and *E. coli* (M3J) (ratio was 1:1:1). The substrate concentration was 100 mM, and final CDW of cells for both reactions was 8 g L^{-1}. Orange line: AA concentration obtained using *E. coli* (M2E_M3J) as catalyst; Blue line: AA concentration obtained using the mixture of *E. coli* (M1D), *E. coli* (M2E) and *E. coli* (M3J) as catalyst. Data are presented as mean value ± SD (standard deviations) of three biological replicates. Source data are provided as a Source Data file.

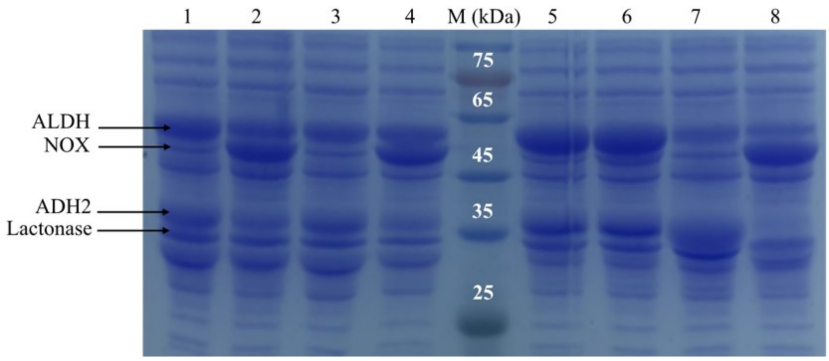

Supplementary Figure 3. SDS-PAGE analysis of whole-cell proteins of Module 3 expressed in *E. coli*. Lane M: protein marker (Mei5 Biotechnology, Co., Ltd); Lane 1: *E. coli* (M3H); Lane 2: *E. coli* (M3J); Lane 3: *E. coli* (M3G); Lane 4: *E. coli* (M3I); Lane 5: *E. coli* (M3B_M3C); Lane 6: *E. coli* (M3B_M3E); Lane 7: *E. coli* (M3A_M3D); Lane 8: *E. coli* (M3A_M3F). Three independent experiments were conducted and similar results were yielded. Source data are provided as a Source Data file.

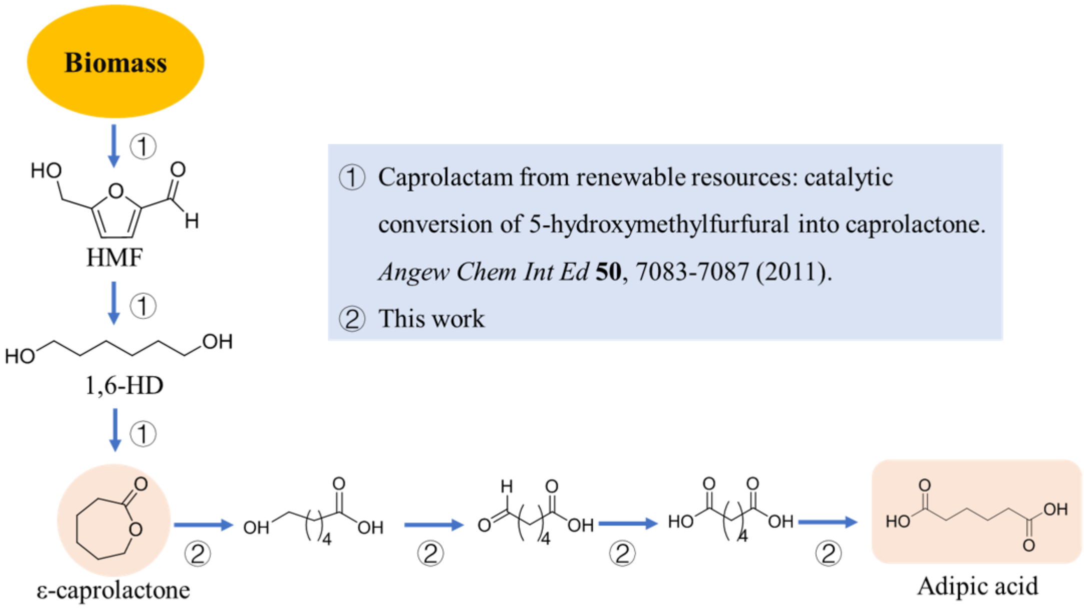

Supplementary Figure 4. Overrall concept for producing adipic acid based on biorenewable feedstock. 5-hydroxymethylfurfural (HMF) can be prepared from biomass. The direct hydrogenation of HMF to 1,6-hexanediol (1,6-HD), then ε-caprolactone obtained *via* dehydrogenation. Finally, ε-caprolactone is converted to adipic acid using modular cell 3 catalyst.

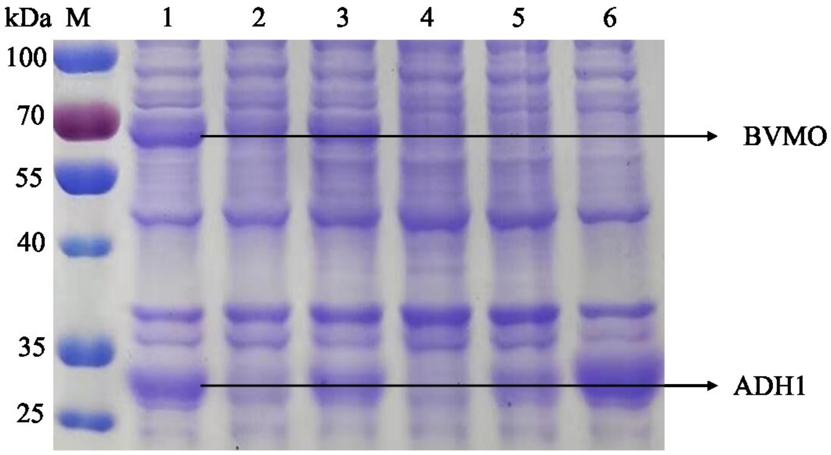

Supplementary Figure 5. SDS-PAGE analysis of whole-cell proteins of Module 2 expressed in *E. coli*. Lane M: protein marker (Thermo Scientific); Lane 1: *E. coli* (M2E); Lane 2: *E. coli* (M2G); Lane 3: *E. coli* (M2F); Lane 4: *E. coli* (M2H); Lane 5: *E. coli* (M2A_M2D); Lane 6: *E. coli* (M2B_M2C). Three independent experiments were conducted and similar results were yielded. Source data are provided as a Source Data file.

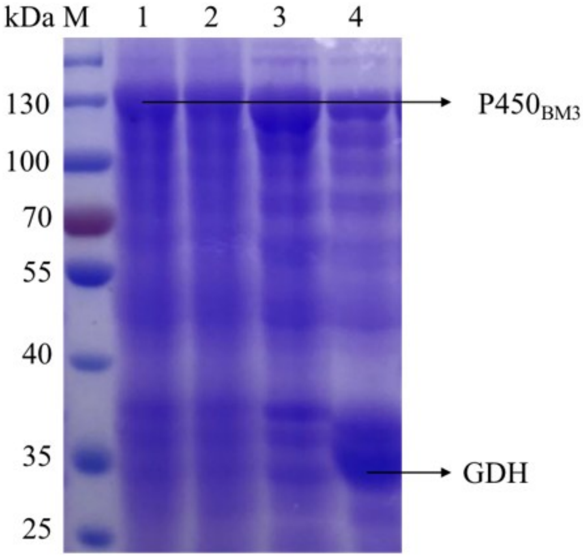

Supplementary Figure 6. SDS-PAGE analysis of whole-cell proteins of Module 1 expressed in *E. coli*. Lane M: protein marker (Thermo Scientific); Lane 1: *E. coli* (M1A); Lane 2: *E. coli* (M1B); Lane 3: *E. coli* (M1C); Lane 4: *E. coli* (M1D). Three independent experiments were conducted and similar results were yielded. Source data are provided as a Source Data file.

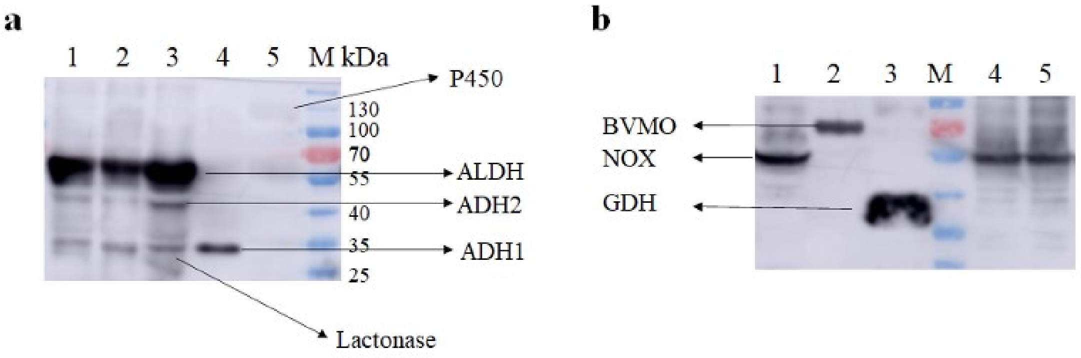

Supplementary Figure 7. **Western blot analysis of recombinant enzyme expressions. a** Hig-tagged enzymes expressed in different strains, lane 1: EC1_2_3, lane 2: EC2_3, lane 3: cell module 3, lane 4: cell module 2, lane 5: cell module 1, M: protein marker (Thermo Scientific). **b** Flag-tagged enzymes expressed in different strains, lane 1: cell module 3, lane 2: cell module 2, lane 3: cell module 1, lane 4: EC1_2_3, lane 5: EC2_3, M: protein marker (Thermo Scientific). The molecular weights of enzymes are shown in Supplementary Table 1. All the samples were supernatants of recombinant *E. coli* after sonication, and for detailed reaction conditions, see Supplementary Methods. Three independent experiments were conducted and similar results were yielded. Source data are provided as a Source Data file.

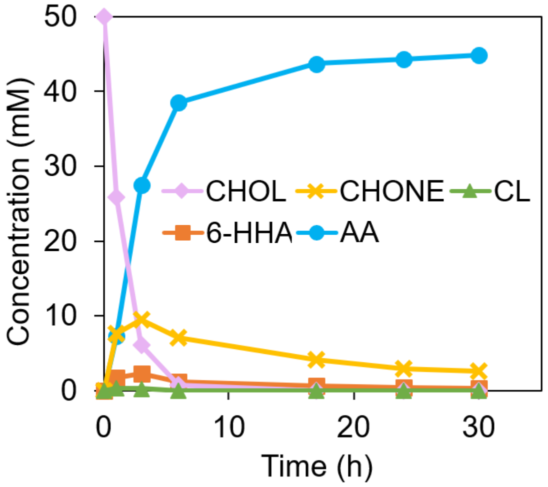

Supplementary Figure 8. Scale-up in bioreactor for converting CHOL 2b to AA 7b. EC2_3-catalyzed conversion of CHOL **2b** (50 mM). For detailed reaction conditions, see Supplementary methods. Purple line: CHOL; yellow line: CHONE; green line: CL; orange line: 6-HHA; blue line:AA. Data are presented as mean value ± SD (standard deviations) of three biological replicates. Source data are provided as a Source Data file.

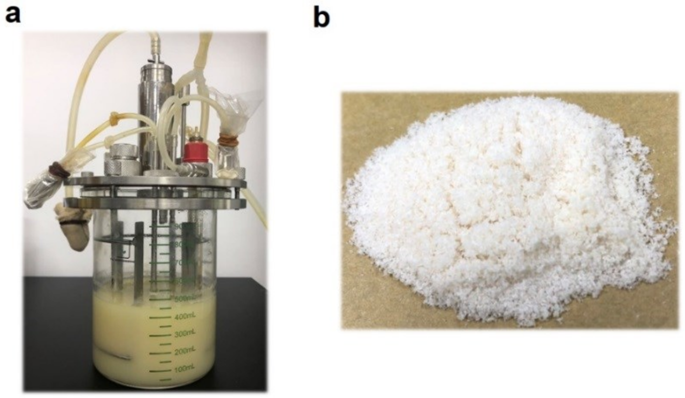

Supplementary Figure 9. Images of the 1-L fermenter and product AA 7b. a 1-L fermenter with reaction mixture for CHOL **2b** bioconversion. **b** The white solid AA **7b** were prepared from the isolation and purification of EC2_3-catalyzed reaction mixture. The yield was 64%.

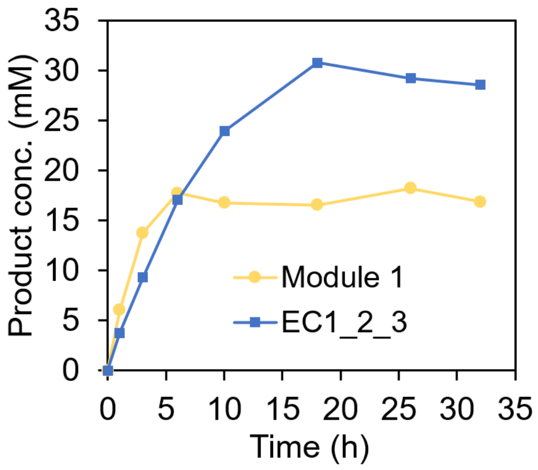

Supplementary Figure 10. Reaction time course with module 1 and EC1_2_3 as catalysts. Cell module 1 converted CH **1b** to CHOL **2b** and CHONE **3b**, and EC1_2_3 converted CH **1b** to AA **7b** under optimized conditions at 100 mM substrate. Yellow line: CHOL concentration obtained using Module 1 as catalyst; blue line: AA concentration obtained using EC1_2_3 as catalyst. Data are presented as mean value from two biological replicates. Source data are provided as a Source Data file.

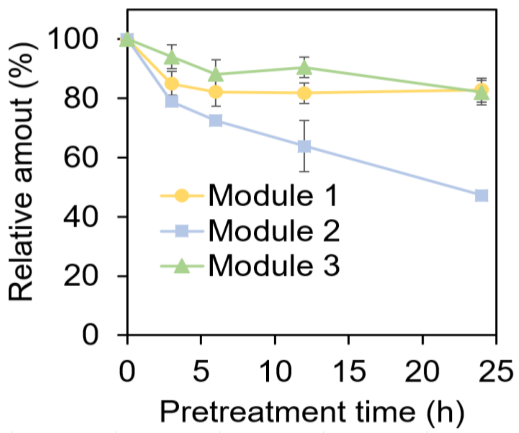

Supplementary Figure 11. Stability assay of each *E. coli* cell module. The cell module (CDW was 8 g L^{-1}) was preincubated at 25°C, 200 r.p.m, afterwards the substrates (final concentration: 100 mM) were added to start the catalytic reactions. The corresponding product concentrations were determined. Yellow line: Module 1; blue line: Module 2; green line: Module 3. Data are presented as mean value ± SD (standard deviations) of three biological replicates. Source data are provided as a Source Data file.

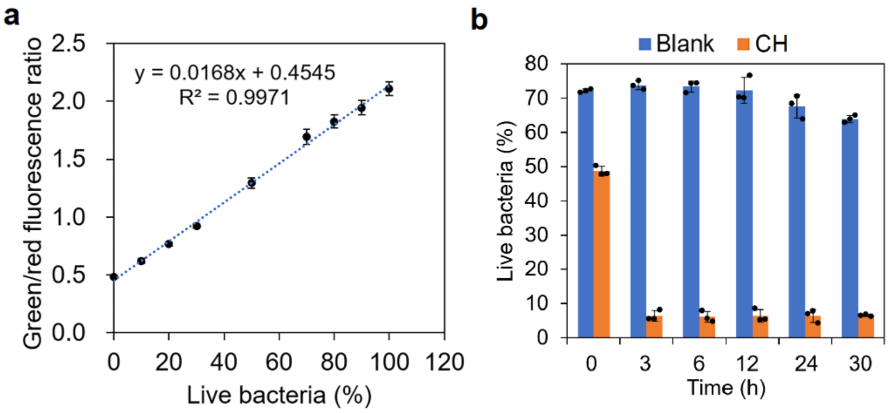

Supplementary Figure 12. Bacterial viability assay during the biocatalytic reaction with EC1_2_3. a standard curve of the assay. **b** the percentage of live *E. coli* cells at different reaction time. Blue column: Blank; orange column: Cyclohexane (CH). The LIVE/DEAD® BacLight™ Bacterial Viability kit was used to measure the proportions of viable *E. coli* cells as described by the manufacturer. Data are presented as mean value ± SD (standard deviations) of three biological replicates. Source data are provided as a Source Data file.

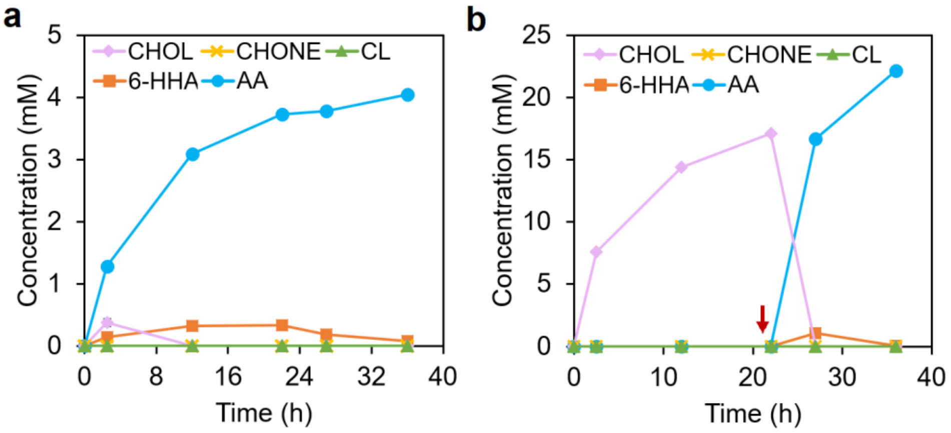

Supplementary Figure 13. Scale-up in bioreactor for converting CH 1b or CHOL 2b to AA 7b. a EC2_3-catalyzed conversion of CHOL **2b** (50 mM). **b** EC1_2_3-catalyzed conversion of CH **1b** (200 mM). **c** CH **1b** (100 mM) was first catalyzed by module 1 (4 g CDW L^{-1}), and then module 2 (8 g CDW L^{-1}) and module 3 (4 g CDW L^{-1}) were added at 22 h of reaction indicated by arrow. For detailed reaction conditions, see Supplementary Methods. Purple line: CHOL; yellow line: CHONE; grreen line: CL; orange line: 6-HHA; blue line:AA. Data are presented as mean value from two biological replicates. Source data are provided as a Source Data file.

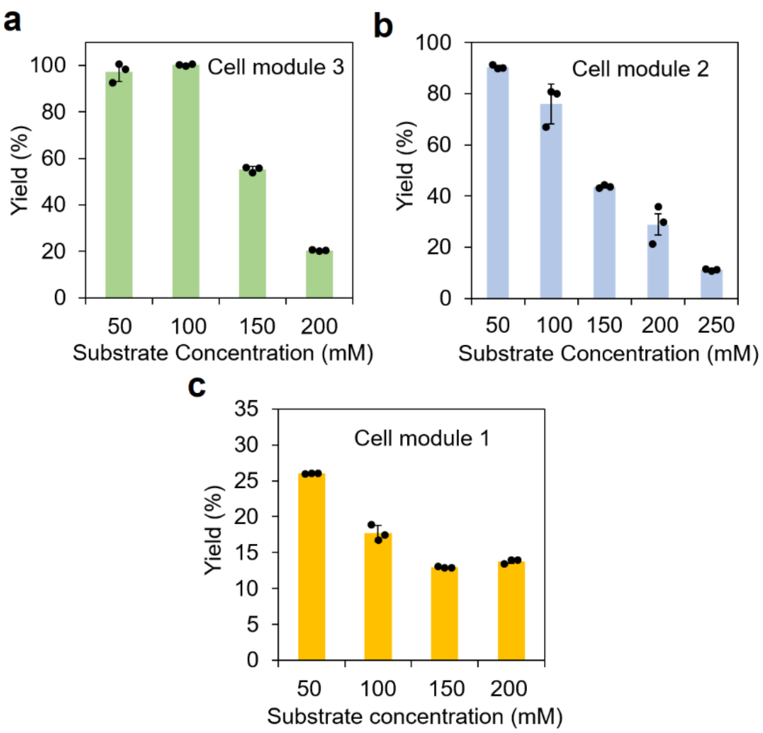

Supplementary Figure 14. Performance of each cell module at varying substrate concentrations. a CL **4b** (50-200 mM) was converted by cell module 3 (*E. coli* (M3B_M3E)) to produce AA **7b**. Green column: yield of AA. **b** CHOL **2b** (50-250 mM) was converted by cell module 2 (*E. coli* (M2E)) to produce CL. Blue column: yield of CL. **c** CH **1b** (50-200 mM) was converted by cell module 1 (*E. coli* (M1D)) to produce CHOL **2b** and CHONE **3b**. Yellow column: yield of CHOL and CHONE. The pH was not controlled during all the reactions. Data are presented as mean value ± SD (standard deviations) of three biological replicates. Source data are provided as a Source Data file.

SH-Rtx-WAX column：

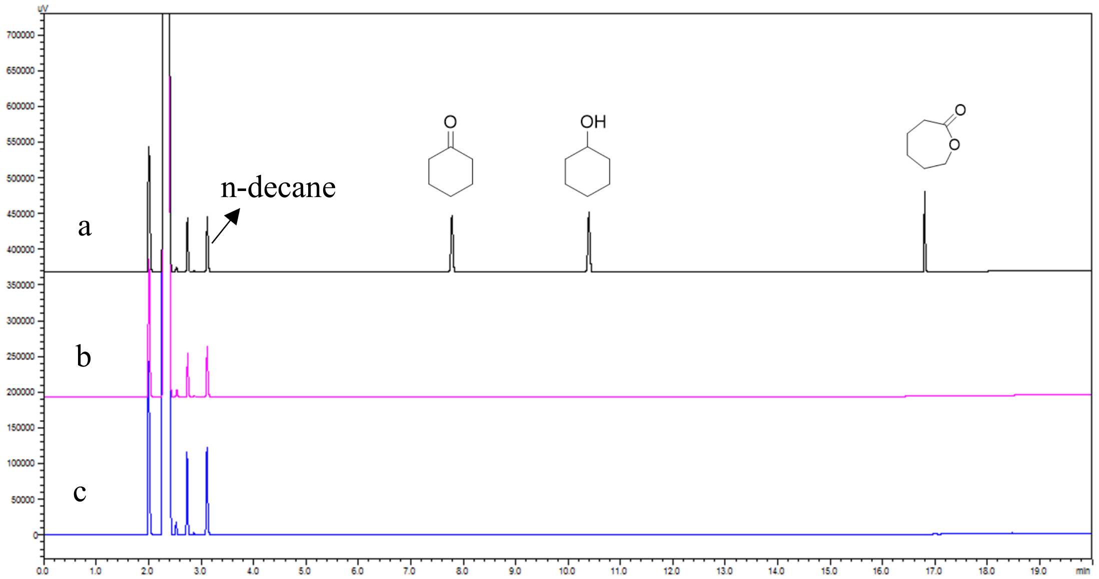

SH-Rtx-1 column：

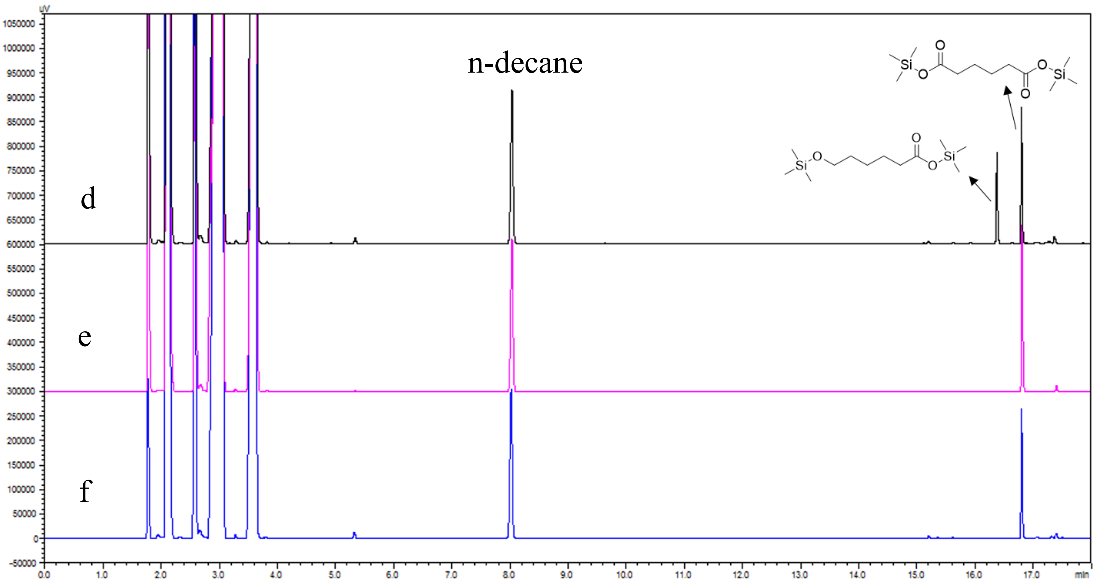

Supplementary Figure 15. **GC analysis of reaction mixture from *E. coli* consortiums catalyzed conversion of either 2b or 1b to 7b.** **a, d** Standards of *n*-decane, cyclohexanol **2b**, cyclohexanone **3b**, ε-caprolactone **4b**, 6-hydroxyhexanoic acid **5b** (6 mM each). **b, e** Representative GC chromatograms of *E. coli* consortium 2_3 catalyzed cascade reactions of cyclohexanol **2b** to adipic acid **7b**. **c, f** Representative GC chromatograms of *E. coli* consortium 1_2_3 catalyzed cascade reactions of cyclohexane **1b** to adipic acid **7b**, there is no intermediates accumulation after reaction.

SH-Rtx-WAX column：

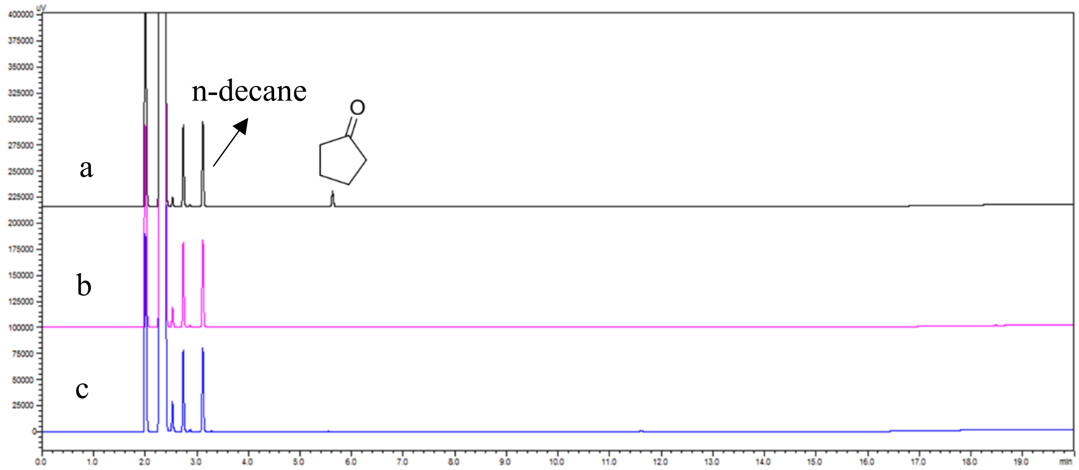

SH-Rtx-1 column：

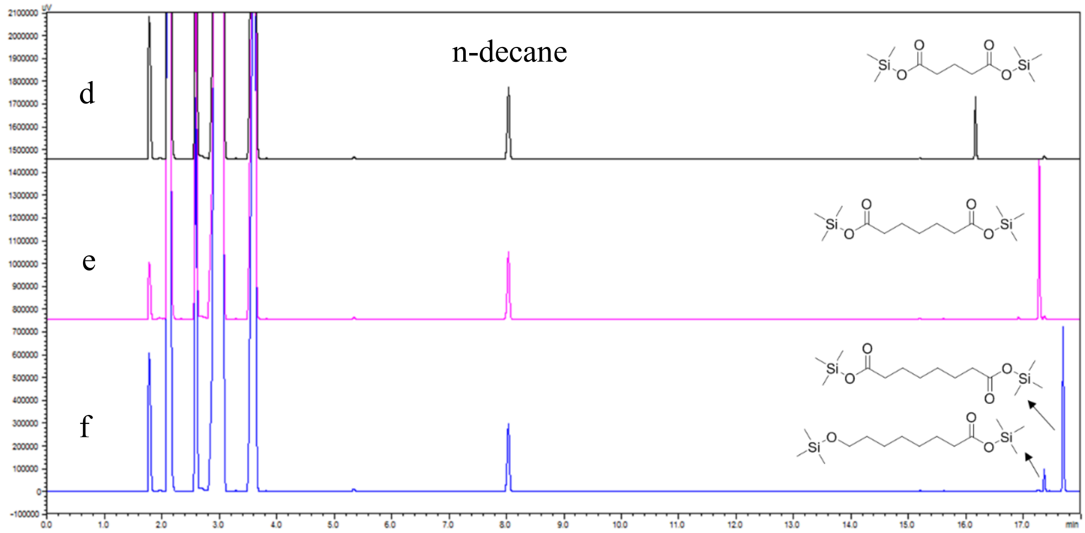

Supplementary Figure 16. **GC analysis of reaction mixtures from *E. coli* consortium 2_3 catalyzed conversion of 2a, 2c, 2d to 7a, 7c, 7d**. **a, d** Representative GC chromatograms of *E. coli* consortium 2_3 catalyzed reactions of cyclopentanol **2a** to glutaric acid **7a**. **b, e** Representative GC chromatograms of *E. coli* consortium 2_3 catalyzed cascade reactions of cycloheptanol **2c** to pimelic acid **7c**. **c**, **f** Representative GC chromatograms of *E. coli* consortium 2_3 catalyzed cascade reactions of cyclooctanol **2d** to octanedioic acid **7d**.

SH-Rtx-WAX column:

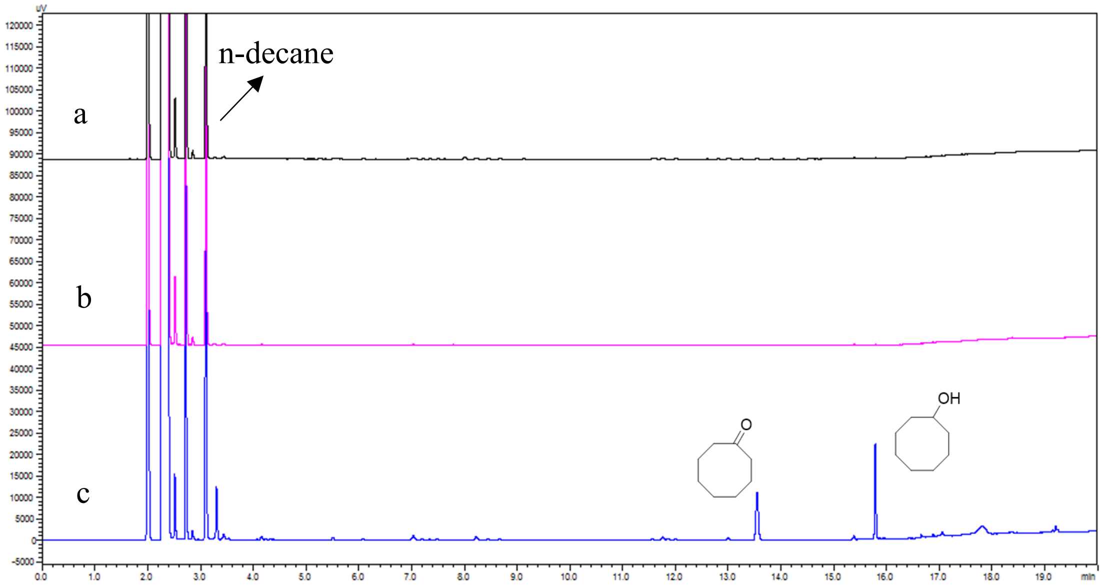

SH-Rtx-1 column:

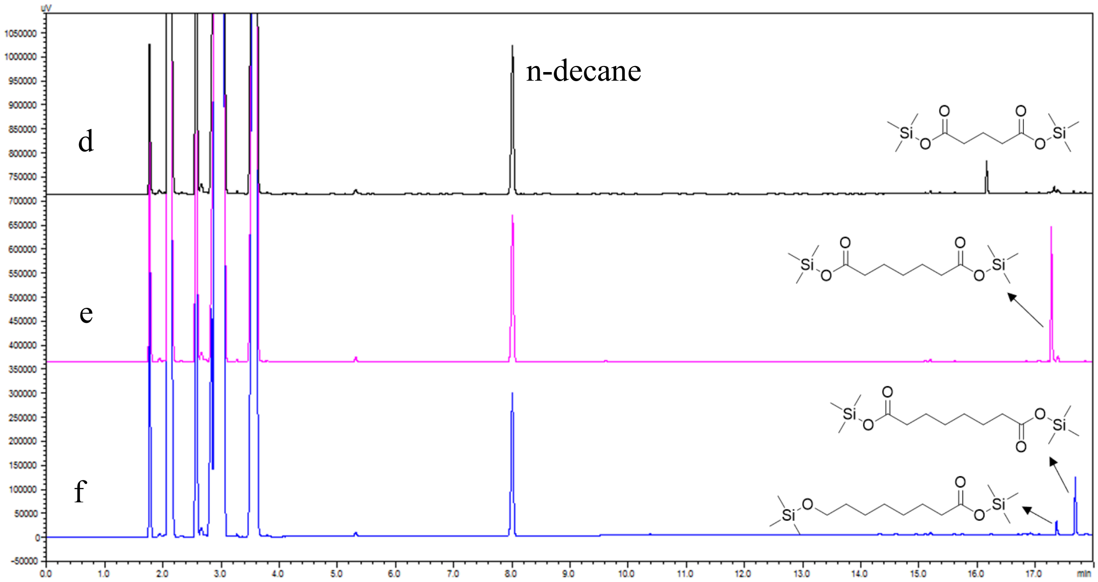

Supplementary Figure 17. **GC analysis of reaction mixtures from *E. coli* consortium 1_2_3 catalyzed converison of 1a, 1c, 1d to 7a, 7c, 7d. a, d** Representative GC chromatograms of *E. coli* consortium 1_2_3 catalyzed reactions of cyclopentane **1a** to glutaric acid **7a**. **b, e** Representative GC chromatograms of *E. coli* consortium 1_2_3 catalyzed cascade reactions of cycloheptane **1c** to pimelic acid **7c**. **c, f** Representative GC chromatograms of *E. coli* consortium 1_2_3 catalyzed cascade reactions of cyclooctane **1d** to octanedioic acid **7d**.

a

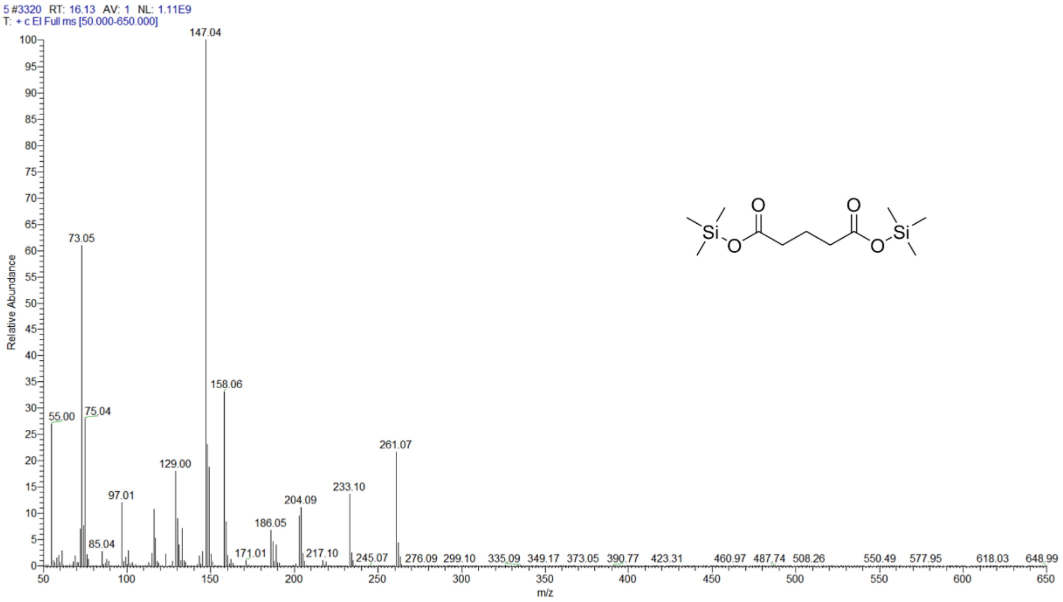

b

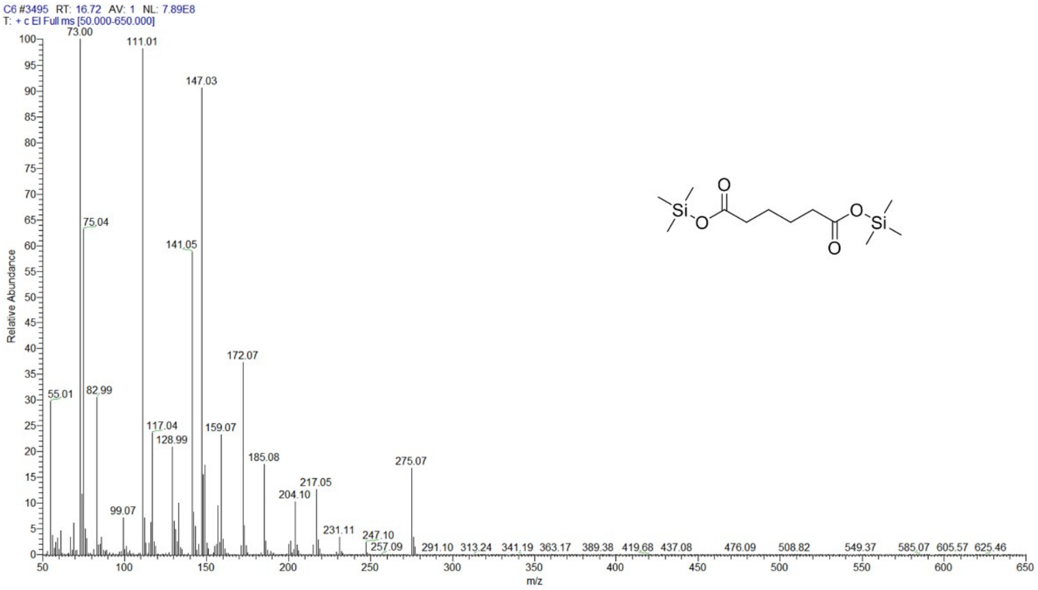

Supplementary Figure 18. **GC-MS analysis of α, ω-dicarboxylic acids 7a-7b**. **a** The fragmentation pattern was obtained for glutaric acid **7a** after derivatization. **b** The fragmentation pattern was obtained for adipic acid **7b** after derivatization. The GC–MS analysis was performed using the Thermo Scientific GC-MS equipped with a DB-5MS column (30 m×0.25 mm, 0.25 μm).

a

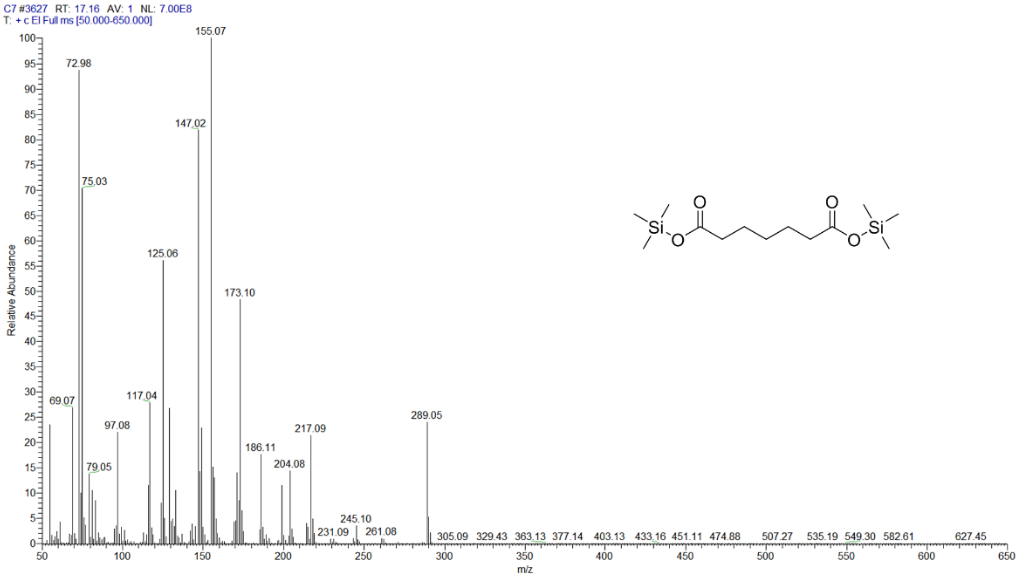

b

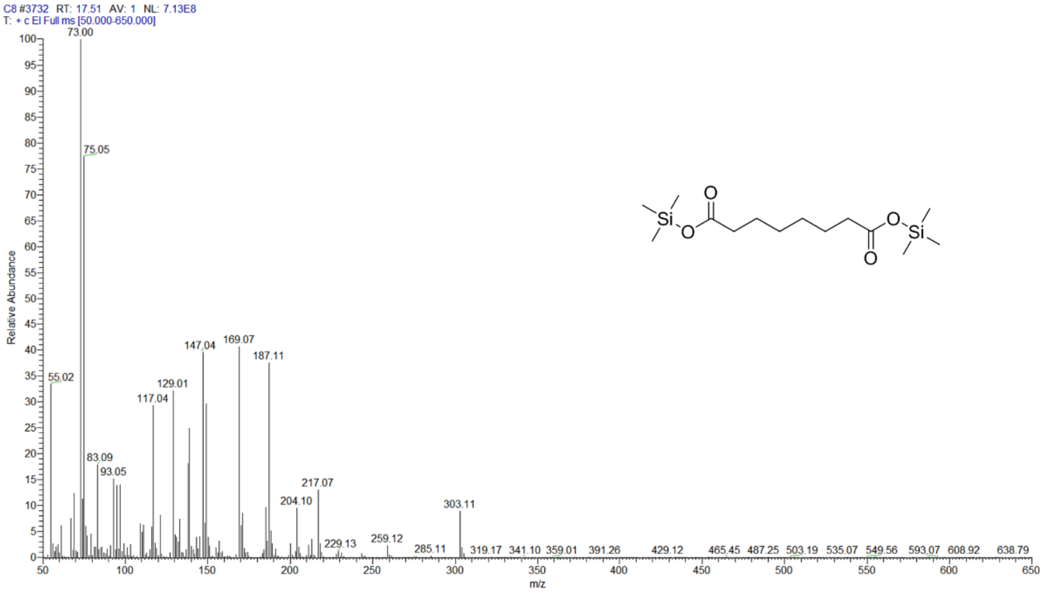

Supplementary Figure 19. **GC-MS analysis of α, ω-dicarboxylic acids 7c-7d**. **a** The fragmentation pattern was obtained for pimelic acid **7c** after derivatization. **b** The fragmentation pattern was obtained for octanedioic acid **7d** after derivatization. The GC–MS analysis was performed using the Thermo Scientific GC-MS equipped with a DB-5MS column (30 m×0.25 mm, 0.25 µm).

a

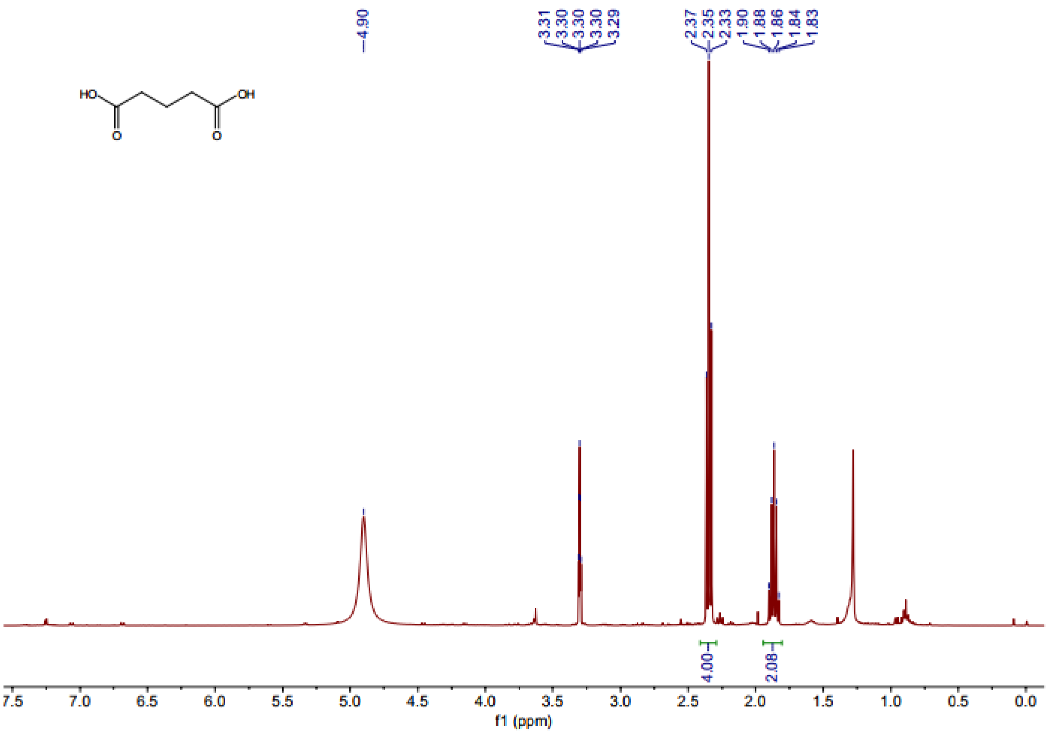

b

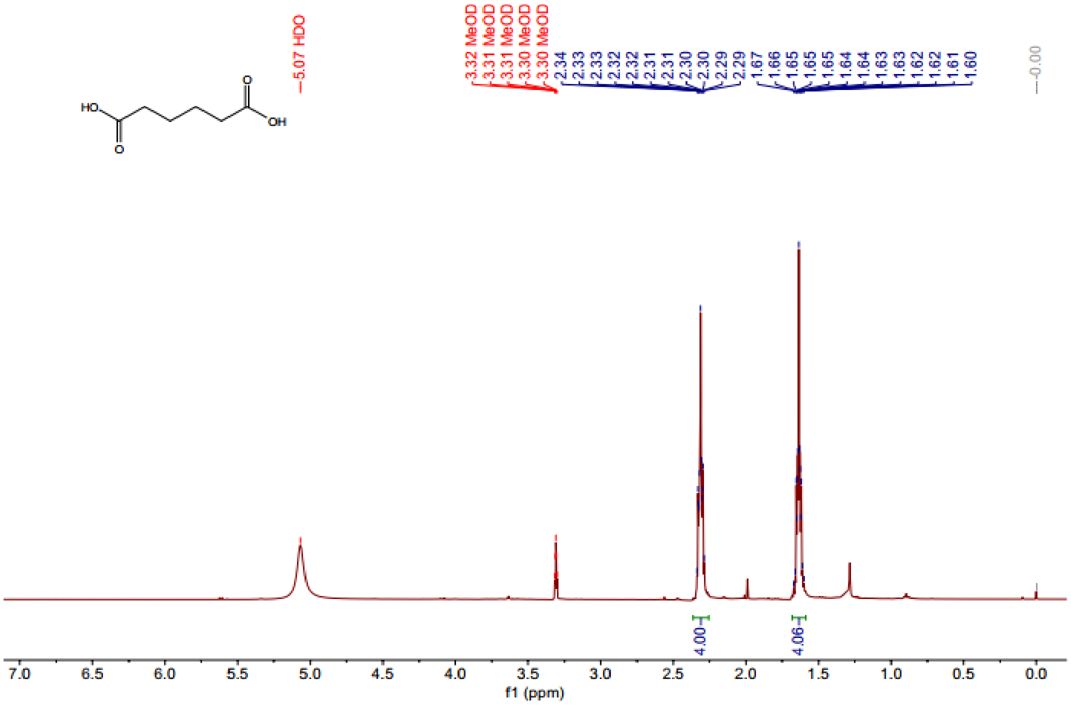

Supplementary Figure 20. **NMR spectra. a** NMR spectrum for **7a**. **b** NMR spectrum for **7b.**

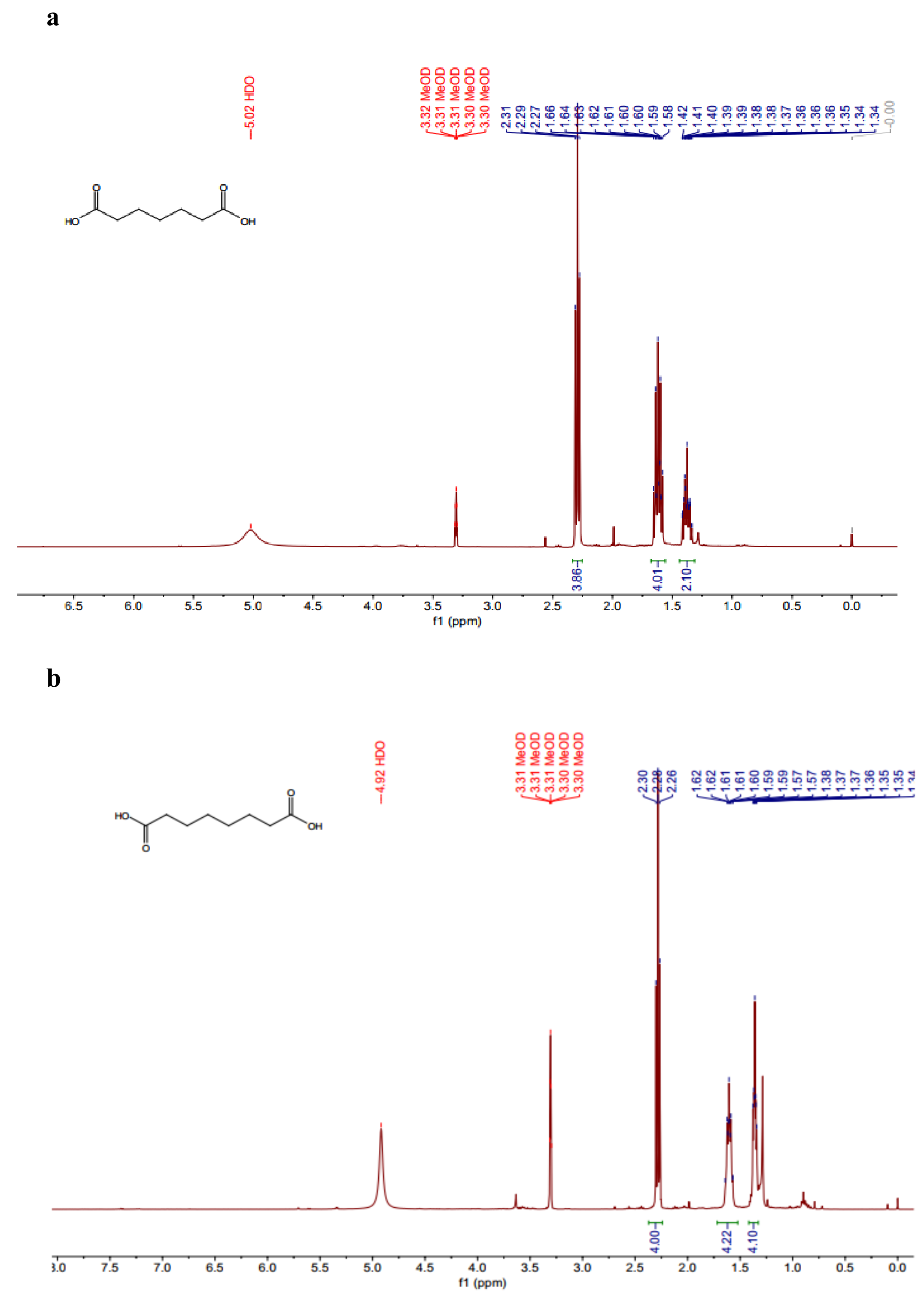

Supplementary Figure 21. **NMR spectra. a** NMR spectrum for **7c**. **b** NMR spectrum for **7d**.

Supplementary References

1. Omura, T. & Sato, R. The carbon monoxide-biding pigment of liver microsomes. *J. Biol. Chem.* **239**, 2370-2378 (1964).
2. Adkins, J., Jordan, J. & Nielsen, D. R. Engineering *Escherichia coli* for renewable production of the 5 - carbon polyamide building - blocks 5 - aminovalerate and glutarate. *Biotechnol. Bioeng.* **110**, 1726-1734 (2011).
3. Zhao, M. et al. Metabolic engineering of *Escherichia coli* for producing adipic acid through the reverse adipate-degradation pathway. *Metab. Eng.* **47**, 254-262 (2018).
4. Clomburg, J. M. et al. Integrated engineering of β-oxidation reversal and ω-oxidation pathways for the synthesis of medium chain ω-functionalized carboxylic acids. *Metab. Eng*. **28**, 202-212 (2015).
